# Unveiling the Virome of Wild Birds: Exploring CRESS-DNA Viral Dark Matter

**DOI:** 10.1093/gbe/evae206

**Published:** 2024-09-27

**Authors:** Ziyuan Dai, Haoning Wang, Juan Xu, Xiang Lu, Ping Ni, Shixing Yang, Quan Shen, Xiaochun Wang, Wang Li, Xiaolong Wang, Chenglin Zhou, Wen Zhang, Tongling Shan

**Affiliations:** Department of Clinical Laboratory, Affiliated Hospital 6 of Nantong University, Yancheng Third People's Hospital, Yancheng, Jiangsu 224000, China; Department of Laboratory Medicine, School of Medicine, Jiangsu University, Zhenjiang, Jiangsu 212013, China; Heilongjiang Cold Region Wetland Ecology and Environment Research Key Laboratory, School of Geography and Tourism, Harbin University, Harbin, Heilongjiang 150086, China; School of Geography and Tourism, Harbin University, Harbin, Heilongjiang 150086, China; Clinical Laboratory Center, The Affiliated Taizhou People's Hospital of Nanjing Medical University, Taizhou 225300, China; Department of Laboratory Medicine, School of Medicine, Jiangsu University, Zhenjiang, Jiangsu 212013, China; Clinical Laboratory Center, The Affiliated Taizhou People's Hospital of Nanjing Medical University, Taizhou 225300, China; Department of Laboratory Medicine, School of Medicine, Jiangsu University, Zhenjiang, Jiangsu 212013, China; Department of Laboratory Medicine, School of Medicine, Jiangsu University, Zhenjiang, Jiangsu 212013, China; Department of Laboratory Medicine, School of Medicine, Jiangsu University, Zhenjiang, Jiangsu 212013, China; Clinical Laboratory Center, The Affiliated Taizhou People's Hospital of Nanjing Medical University, Taizhou 225300, China; The Key Laboratory of Wildlife Diseases and Biosecurity Management of Heilongjiang Province, Northeast Forestry University, Harbin 150006, China; Clinical Laboratory Center, The Affiliated Taizhou People's Hospital of Nanjing Medical University, Taizhou 225300, China; Department of Laboratory Medicine, School of Medicine, Jiangsu University, Zhenjiang, Jiangsu 212013, China; Shanghai Veterinary Research Institute, Chinese Academy of Agricultural Sciences, Shanghai 200241, China

**Keywords:** metagenomic, Cressdnaviricota, wild bird, dark matter

## Abstract

Amid global health concerns and the constant threat of zoonotic diseases, this study delves into the diversity of circular replicase-encoding single-stranded DNA (CRESS-DNA) viruses within Chinese wild bird populations. Employing viral metagenomics to tackle the challenge of “viral dark matter,” the research collected and analyzed 3,404 cloacal swab specimens across 26 bird families. Metagenomic analysis uncovered a rich viral landscape, with 67.48% of reads classified as viral dark matter, spanning multiple taxonomic levels. Notably, certain viral families exhibited host-specific abundance patterns, with Galliformes displaying the highest diversity. Diversity analysis categorized samples into distinct groups, revealing significant differences in viral community structure, particularly noting higher diversity in terrestrial birds compared to songbirds and unique diversity in migratory birds versus perching birds. The identification of ten novel Circoviridae viruses, seven Smacoviridae viruses, and 167 Genomoviridae viruses, along with 100 unclassified CRESS-DNA viruses, underscores the expansion of knowledge on avian-associated circular DNA viruses. Phylogenetic and structural analyses of Rep proteins offered insights into evolutionary relationships and potential functional variations among CRESS-DNA viruses. In conclusion, this study significantly enhances our understanding of the avian virome, shedding light on the intricate relationships between viral communities and host characteristics in Chinese wild bird populations. The diverse array of CRESS-DNA viruses discovered opens avenues for future research into viral evolution, spread factors, and potential ecosystem impacts.

**Significance**

This study uncovers 284 new genomic sequences of CRESS-DNA viruses, shedding light on the previously unknown diversity within this viral phylum. By analyzing wild and breeding bird specimens, this research provides valuable insights into the presence and diversity of CRESS-DNA viruses in avian populations, expanding our understanding of viral ecology in birds. This dataset serves as a foundational resource for future studies investigating the evolution, genetic diversity, and ecological significance of CRESS-DNA viruses, facilitating deeper exploration into their biology and potential implications.

## Introduction

With technological advances and the development of economic globalization, human–wildlife interaction has become increasingly common, significantly increasing the likelihood of zoonotic pathogens spilling over into human populations. Birds, with their strong flying abilities and vast geographic distribution, pose significant risks for the spread and transmission of infectious diseases. It is critical to note that wild migratory birds connect different parts of the world through their annual cyclic migration (Brook et al. 2020). A significant number of wild birds migrate across national and continental borders, serving as intermediate hosts or carriers of pathogens. As they migrate, they transmit these pathogens to other birds, poultry, and livestock, and even to humans through direct contact and vectors such as air, food, and water (Olsen et al. 2006; Wille and Holmes 2020). Since the 20th century, bird diseases such as West Nile fever, avian influenza, and avian cholera have frequently emerged, causing numerous deaths among wild birds, poultry, and even humans, and having a severe impact on society.

When researchers explore new viral genomes within complex microbial communities associated with humans and other animals, they often discover a much larger number of sequences than anticipated. However, a significant portion of these detected viral sequences (60% to 95%) (Roux et al. 2015) is categorized as “viral dark matter” due to their low similarity to reference sequences or the lack of functional and classification annotations (Krishnamurthy and Wang 2017). This “viral dark matter” poses a major obstacle to studying viruses comprehensively. Recent studies have indicated that our understanding of viral diversity, including known viruses directly infecting humans, is incomplete (Turnbaugh et al. 2007; Pastrana et al. 2018). To achieve a more comprehensive classification of unknown viral groups and explore their associations with diseases, it is necessary to continuously identify, annotate, and publicly share the obtained viral genomes. Viral metagenomics, combined with next-generation sequencing (NGS) technologies, enables the mining of a large number of viral dark matter sequences without the need for isolation or cultivation, providing a new systematic approach for the analysis and identification of partially low abundance and highly diverse viruses. China possesses abundant biological resources, presenting significant potential for the exploration of viral dark matter.

Circular replicase (Rep)-encoding single-stranded DNA (ssDNA) viruses, known as CRESS-DNA viruses, members of phylum Cressdnaviricota, were first described by Rosario, Duffy, et al. (2012) to include a group of viruses that possessed circular ssDNA genomes and encode an enzyme responsible for initiating genome replication (Krupovic et al. 2020). These viruses are highly prevalent and infect species from all three domains of life (Archaea, Prokarya, and Eukarya) (Gutierrez 1999; Wawrzyniak et al. 2017; Díez-Villaseñor and Rodriguez-Valera 2019; Kazlauskas et al. 2019; Tarasova et al. 2021). CRESS-DNA viral genomes vary from 1.0 to 25 kb (Mochizuki et al. 2012; Briddon et al. 2018). The Rep protein is thought to initiate replication through the rolling circle replication (RCR) mechanism.

Over the past few decades, viral metagenomic studies have expanded the number of newly described, often unclassified, viruses within the phylum Cressdnaviricota (Kaszab et al. 2018; Moens et al. 2018; Kaszab et al. 2020; Capozza et al. 2022). The phylum comprises two classes: the class Repensiviricetes, currently encompassing all plant and fungal viruses of the families Genomoviridae and Geminiviridae, and the class Arfiviricetes, which incorporates ten virus families (Bacilladnaviridae, Circoviridae, Vilyaviridae, Smacoviridae, Amesuviridae, Nanoviridae, Metaxyviridae, Redondoviridae, Naryaviridae, and Nenyaviridae). However, numerous groups of related ssDNA viruses, informally denoted as CRESSV1 to CRESSV6, have remained unclassified (Kazlauskas et al. 2018; Krupovic et al. 2020). Association with bird-related samples and human infections has been reported for these viruses, although their pathogenic role remains unverified to date. Despite recent publications on CRESS-DNA viruses harbored by birds, additional sequence data from diverse geographic regions are required to ascertain the evolutionary origins and divergence of these viruses. This study presents the first data set on CRESS-DNA viruses circulating among birds in China.

In the future, the emergence of newly infectious diseases originating from animals will become a “new normal” that humans must face. Conducting research on the prevention and control of zoonotic and emerging infectious diseases, using viral metagenomics and NGS technologies to analyze the viral community in wild animals, can not only reveal the baseline information of the viral community in wild animals but also, more importantly, enable us to analyze and identify new viruses in depth, predict potential new viral infections in the future.

## Results

### Overview of Virome

**Fig. 1.**
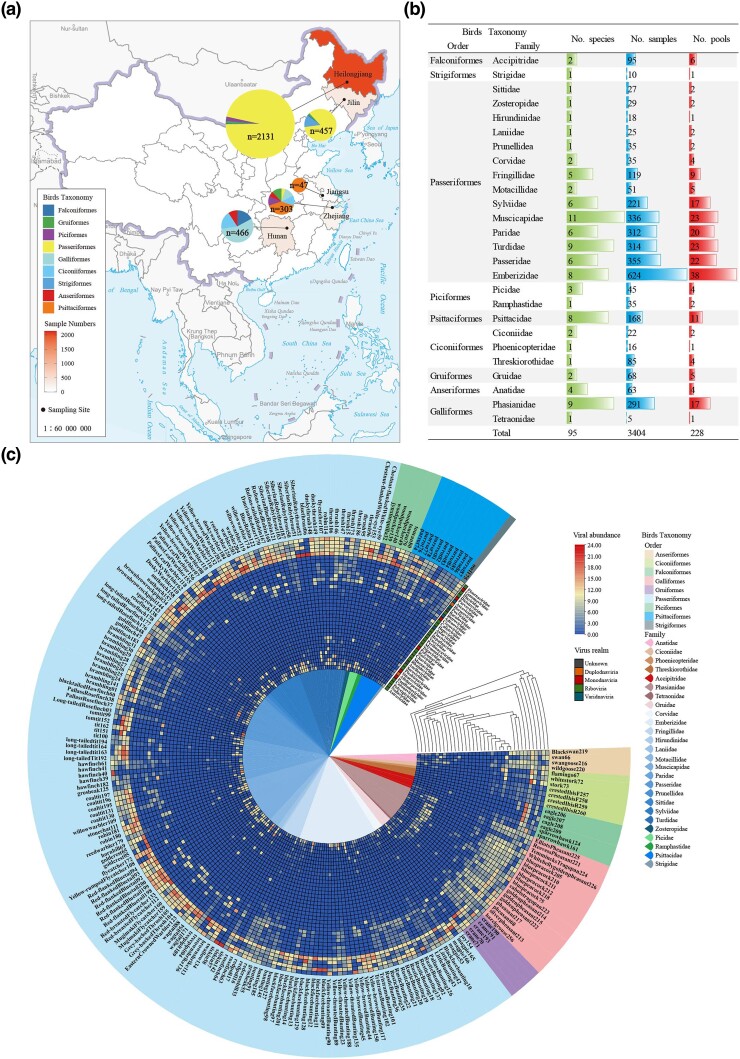
Sample collection and DNA library construction. a) Map of sampling locations. The sampling sites are marked with colors. Samples were obtained from 5 provinces in China: Heilongjiang, Jilin, Zhejiang, Jiangsu, and Hunan. b) Overview of avian taxa and library construction status. The avian taxa include 9 orders (Falconiformes, Gruiformes, Coraciiformes, Passeriformes, Anseriformes, Galliformes, Ciconiiformes, Strigiformes, and Psittaciformes) comprising 26 families, with a cumulative total of 95 species. The bars represent the number of avian species, samples, and pools for each avian family. c) Distribution of 36 viral families in 228 pools. The color scale represented the relative abundance levels. The shades ranging from red to blue indicated the relative abundance, with red representing higher abundance and blue representing lower abundance.

This study included 3,404 wild bird cloacal swab specimens belonging to 26 different families of birds. The 3,404 samples were combined into 228 pools based on collection time, collection location, and bird species for viral metagenomic analysis (Fig. 1a and b). Each pool consists of an average of 15 samples (supplementary table S1, Supplementary Material online). After Illumina sequencing, a total of 46,494,515 reads showing similarity to viruses were obtained, accounting for 9.62% of the total reads. The cellular organisms (archaea, bacteria, and eukaryotes) and other nonvirion-associated reads were removed. Species accumulation analysis revealed that our population captured most viral species from bird cloacal (supplementary fig. S1, Supplementary Material online). Among them, 15,119,231 reads can be effectively classified and annotated at the *genus* level in the current ICTV classification system. The remaining 31,375,284 reads (approximately 67.48%) are considered *viral dark matter*.

A total of 4 viral domains (Duplodnaviria, Monodnaviria, Riboviria, and Varidnaviria), 5 kingdoms, 10 phyla, 19 classes, 26 orders, 36 families, and 158 genera were detected in the cloacal swab samples of birds. A taxonomy tree generated by GraPhlAn was utilized to expedite the identification of dominant taxa within the intricate viral data. As shown in supplementary fig. S2, Supplementary Material online, the colored pentagon nodes represented the hosts of 36 viral families in the tree, encompassing vertebrates, invertebrates, plants, fungi, and bacteria. The three most abundant phyla were Pisuviricota (50.31% of the total sequences), Cossaviricota (30.28%), and Cressdnaviricota (4.01%), together accounting for 84.60% of the total sequences. The most prevalent families were Parvoviridae (30.26%), Dicistroviridae (21.03%), Picornaviridae (14.33%), Iflaviridae (10.75%), Genomoviridae (3.78%), and Astroviridae (1.77%) together comprising 81.92% of the total sequences (supplementary fig. S3, Supplementary Material online). Furthermore, it was evident from Fig. 1c that these six viral families exhibited higher abundance across all pools. The families Astroviridae, Polycipiviridae, and Nodaviridae were relatively abundant in pools from the Passeriformes and Columbiformes orders, while they showed lower abundance in libraries from the Ciconiiformes, Falconiformes, and Psittaciformes orders. Overall, the 17 pools from the Galliformes order displayed a diverse range of viral species, followed by the Ciconiiformes and Anseriformes orders, while the pools of Falconiformes exhibited the lowest number and abundance of viral species.

### Diversity Analysis of Viral Community Structure

**Fig. 2.**
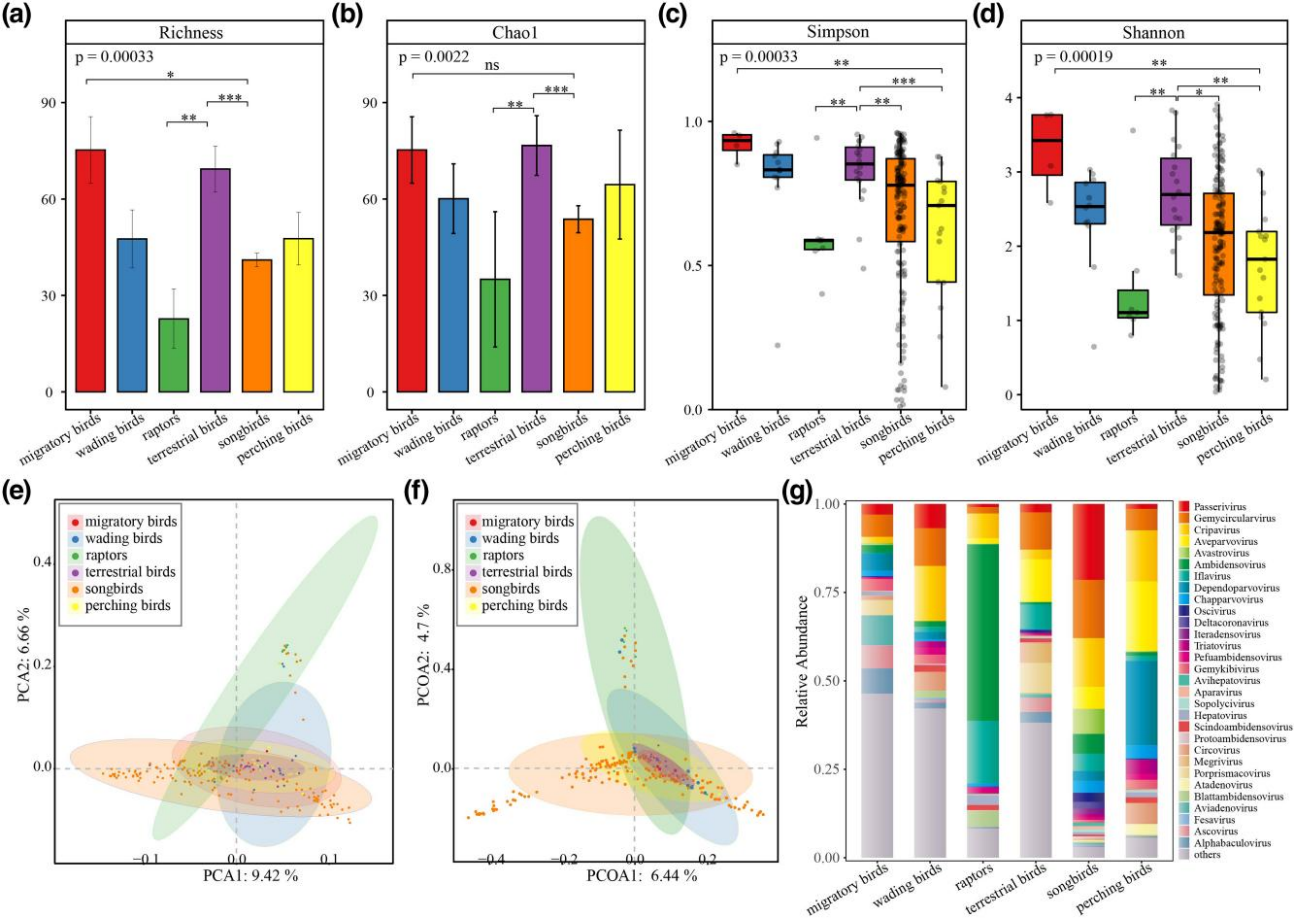
Diversity analysis of viruses in the intestinal tract of birds. a to d) Richeness, Chao1, Simpson, and Shannon index difference analysis. *P < 0.05, **P < 0.01, ***P < 0.001, between the two groups. e) PCA analysis. f) PCOA analysis. g) Stack bar diagram.

Based on the different virus hosts and their distributions, combined with the variations in the dietary preferences and habits of different avian species, the 228 pools were classified into 6 groups: migratory birds, terrestrial birds, wading birds, perching birds, raptors, and songbirds. To further understand the similarities and differences of viral communities within samples and among different groups, diversity analysis was conducted. Alpha diversity indices, including Shannon *H*, Simpson (1 − *D*), richness, Chao1, inverse Simpson, Pielou, and Good’s coverage, are presented in Table 1, while the differences in diversity indices are shown in Fig. 2a to d. Although the group of songbirds exhibited a much higher number of viral species compared to the group of terrestrial birds, the diversity indices of Shannon *H*, Simpson (1 − *D*), richness, and Chao1 in the group of terrestrial birds were higher than those in the group of songbirds. The differences between the two groups were statistically significant according to the Wilcoxon rank-sum test (*P* < 0.05). Additionally, the diversity indices of the group of songbirds were significantly higher than those of the group of raptors (*P* < 0.01). There were significant differences in Shannon *H* and Simpson (1 − *D*) diversity indices between the group of migratory birds and the group of perching birds (3.30 vs. 1.75, 0.92 vs. 0.62, *P* < 0.05). This indicates that the diversity of the group of songbirds, raptors, and perching birds is lower compared to the group of migratory birds and terrestrial birds. The Good’s coverage indices for each group were all above 0.94, suggesting that the current sequencing depth is sufficient to saturate the diversity of avian intestinal viruses.

**Table 1:** Alpha diversity index of viruses in the intestinal tract of birds in each group

Group	Richness		Chao1		Shannon *H*		Simpson (1 − *D*)		Inverse Simpson		Pielou		Good's coverage	
	Mean	SE	Mean	SE	Mean	SE	Mean	SE	Mean	SE	Mean	SE	Mean	SE
Migratory birds	75.25	10.35	75.25	10.35	3.30	0.29	0.92	0.02	16.02	4.11	0.53	0.03	1.00	0.00
Wading birds	47.58	9.02	60.11	10.82	2.39	0.19	0.80	0.05	7.11	1.04	0.46	0.04	0.94	0.03
Raptors	22.71	9.22	35.01	21.04	1.48	0.36	0.60	0.06	4.43	2.18	0.35	0.04	0.98	0.02
Terrestrial birds	69.33	7.17	76.64	9.28	2.74	0.15	0.83	0.03	8.71	1.34	0.45	0.02	0.99	0.01
Songbirds	41.05	2.14	53.72	4.20	2.01	0.07	0.68	0.02	5.90	0.39	0.39	0.01	0.98	0.00
Perching birds	47.65	8.17	64.47	16.93	1.75	0.20	0.62	0.06	3.73	0.54	0.33	0.03	0.99	0.00

To gain insight into similarities in the viral community structures among the six groups, principal component analysis (PCA) of beta diversity analysis was performed based on the Euclidean distances, which demonstrated certain differences in the community structure among the groups. As shown in Fig. 2e, the community structure exhibited some separation between groups, such as the group of raptors, songbirds, and wading birds, while the group of terrestrial birds and migratory birds showed some overlap. The first two principal components accounted for 9.42% and 6.66% of the total variation, respectively. Similarly, principal coordinate analysis (PCoA) based on Bray–Curtis distance yielded similar results (Fig. 2f). Analysis of similarity indicated an *R*-value of 0.095, which was greater than the 99th percentile (0.0935) of the null model, and a *P* < 0.001, indicating significant differences between groups that exceeded within-group differences, thus confirming the effectiveness of the grouping. Figure 2g illustrates the distributional differences of viral genera at the family level among the six groups of bird species. The group of migratory birds was predominantly characterized by *Aviadenovirus*, *Alphabaculovirus*, *Ascovirus*, and *Gemycircularvirus*. The group of wading birds exhibited relatively high abundance of *Cripavirus* and *Gemycircularvirus*, along with *Asfivirus* and *Parapoxvirus*; however, the latter two viruses were relatively low in abundance across all pools and were not displayed in the stacked bar chart. The group of raptors showed relatively high abundance of *Ambidensovirus*, *Iflavirus*, *Cripavirus*, and *Blattambidensovirus*. The group of terrestrial birds exhibited relatively high abundance of *Aveparvovirus*, *Gemycircularvirus*, *Porprismacovirus*, *Iflavirus*, *Megrivirus*, and *Ascovirus*. The group of songbirds displayed high viral abundance, with the six most abundant genera being *Passerivirus*, *Gemycircularvirus*, *Cripavirus*, *Avastrovirus*, *Aveparvovirus*, and *Ambidensovirus*. The group of perching birds was primarily characterized by *Dependoparvovirus*, *Aveparvovirus*, *Cripavirus*, *Gemycircularvirus*, *Circovirus*, *Triatovirus*, *Chapparvovirus*, and *Atadenovirus*.

### Identification of Novel Viruses of the Family Circoviridae

The family Circoviridae is comprised of viruses with circular, covalently closed, ssDNA genomes (approximately 1.7 to 2.4 kb in length) (Breitbart et al. 2017). An ambisense structure was observed in their genomes, featuring two major open reading frames (ORFs), namely the replication-associated protein (rep) gene and the capsid protein (cap) gene (Delwart and Li, 2012). Members of the family are classified into two genera, *Circovirus* and *Cyclovirus*, with the species demarcation threshold of 80% genome-wide nucleotide sequence identity (Breitbart et al. 2017; Dai et al. 2021).

**Fig. 3.**
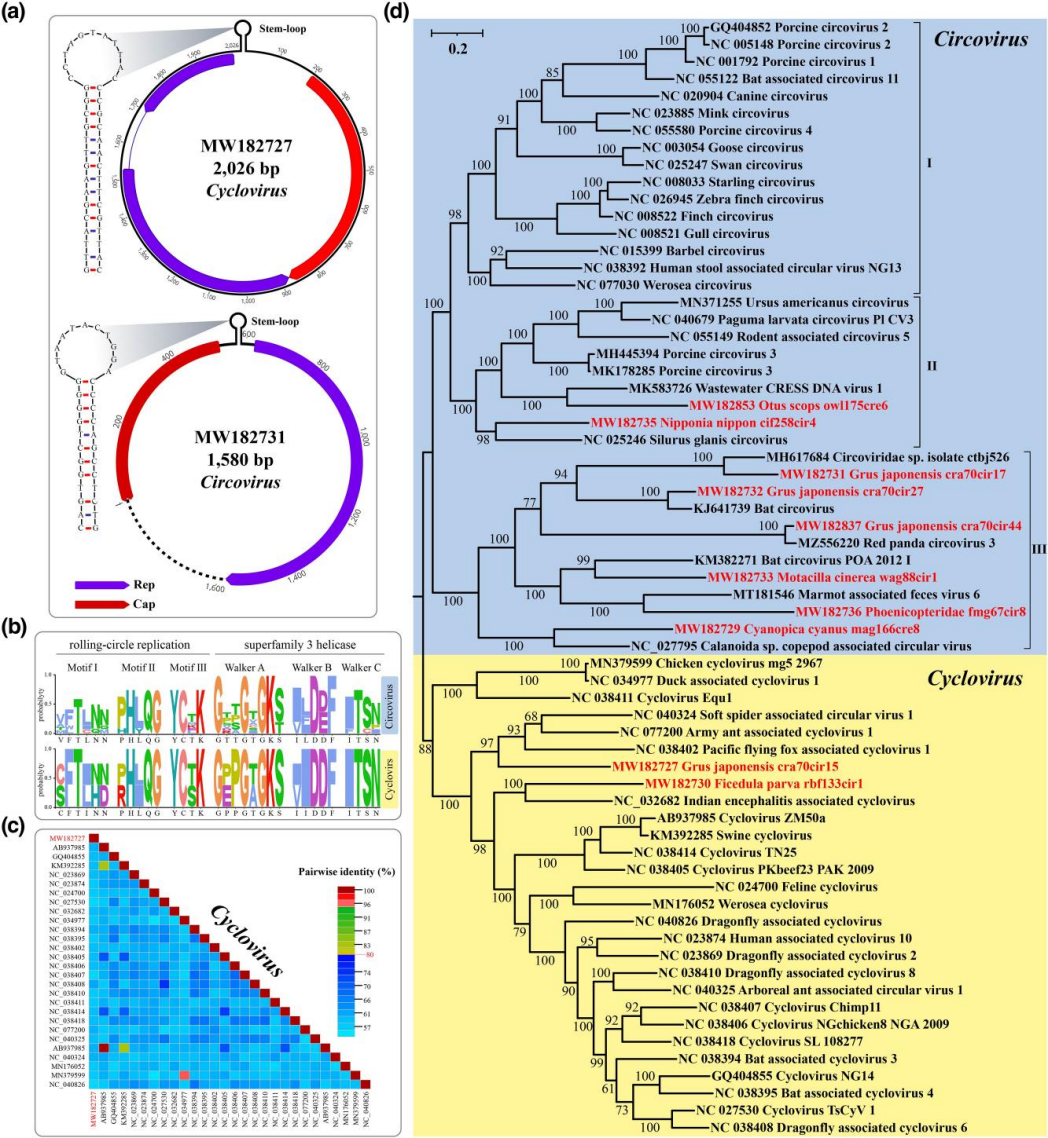
Identification of novel viruses of the family Circoviridae. a) Predicted genome organization of MW182727 and MW182731. b) Identification of the RCR domain and superfamily 3 helicase domain in the Rep protein. c) Pairwise sequence comparison produced with full-genome sequences of cycloviruses within Bayesian consensus tree. The novel cyclovirus is highlighted. d) Bayesian inference tree was constructed using MrBayes v3.2 based on amino acid sequences of Rep protein and visualized with iTOL. The viruses identified in this study are highlighted within the tree. Scale bar indicates the amino acid substitutions per site.

We identified a total of 10 novel viruses of the Circoviridae, including 2 belonging to the genus *Cyclovirus* and 8 belonging to the genus *Circovirus*. The genome lengths of the identified members ranged from 939 to 2,369 bp, with an average of 1,493 bp and an average GC content of 46.38%. As shown in Fig. 3a, the full genome of cra70cir15 (accession number: MW182727), belonging to the genus *Cyclovirus*, was 2,026 bp in length. A putative stem-loop structure containing a highly conserved nine-nucleotide (TAGTATTAC) is considered the origin of replication and was identified at the intergenic region between the 5′-ends of the Cap and Rep ORFs. The cap gene (225 amino acids) was encoded on the same strand as the stem-loop, while the rep gene (222 amino acids) was encoded on the opposite strand. The intergenic region between these two genes was short, comprising only 2 bp. A full-genome sequence similarity analysis was conducted on cra70cir15, revealing an identity range of 55.40% to 61.17% with members of *Cyclovirus*, which fell below the species demarcation threshold of 80% (Fig. 3c). As a result, it was determined that cra70cir15 represents a novel viral species. Within the genus *Circovirus*, cra70cir17 (accession number: MW182731) encoded a replication protein (318 amino acids) on the same strand as the stem-loop, while a capsid protein larger than 176 amino acids was encoded on the opposite strand. Similar to known circoviruses, the conserved RCR motifs and S3H motifs of the Rep proteins in the novel viruses were identified (Fig. 3b).

Based on phylogenetic analysis of the Rep protein (Fig. 3d), it was observed that the two novel viruses belonging to the genus *Cyclovirus* clustered together with circoviruses identified from dragonflies and bats. The genus *Circovirus* shows three separate clusters: one contained the Porcine circovirus 1 (PCV1), Porcine circovirus 2 (PCV2), and Porcine circovirus 4 (PCV4); the second comprised Porcine circovirus 3 (PCV3); and the third was composed of Bat circovirus and Red panda circovirus 3. Although the eight newly identified circoviruses belonging to the genus *Circovirus* were found in avian hosts, they did not cluster with previously known avian-associated circoviruses on the evolutionary tree. Instead, they clustered with circoviruses identified in European perch, ground squirrels, red pandas, bats, algae, and sewage. Except for cra70cir27 (accession number: MW182732) and cra70cir44 (accession number: MW182837), which showed a sequence similarity of over 85% with known viral protein sequences in GenBank, the remaining viruses exhibited less than 70% sequence similarity (supplementary table S2, Supplementary Material online).

### Identification of Novel Viruses of the Family Smacoviridae

The members of the family Smacoviridae possess a small circular ssDNA genome, approximately 2.3 to 3.0 kb in length, containing two major ORFs, encoding typical Rep and the Cap proteins. Currently, the family Smacoviridae is divided into 12 genera, *Babosmacovirus*, *Bonzesmacovirus*, *Bostasmacovirus*, *Bovismacovirus*, *Cosmacovirus*, *Dragsmacovirus*, *Drosmacovirus*, *Felismacovirus*, *Huchismacovirus*, *Inpeasmacovirus*, *Porprismacovirus*, and *Simismacovirus*. The proposed genus-level demarcation threshold for members of the family Smacoviridae is 40% amino acid sequence identity in the Rep protein, accompanied by strong phylogenetic support. Additionally, the recommended species demarcation threshold is 77% genome-wide nucleotide sequence identity (Varsani and Krupovic 2018).

**Fig. 4.**
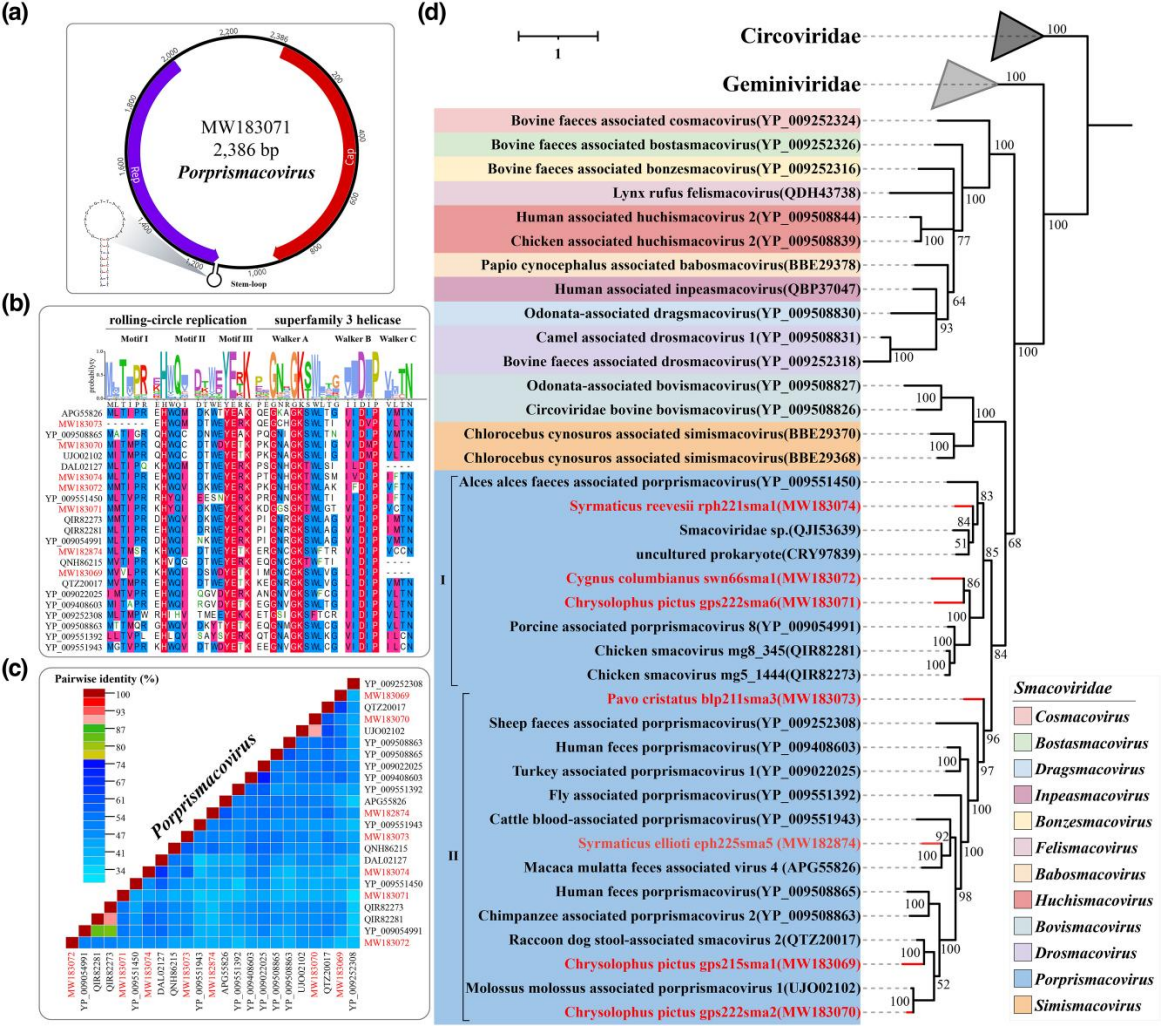
Identification of novel viruses of the family Smacoviridae. a) Predicted genome organization of MW183071. b) Identification of the rolling circle replication domain and superfamily 3 helicase domain in the Rep protein. c) Pairwise sequence comparison produced with amino acid sequences of Rep protein of porprismacoviruses within Bayesian consensus tree. The novel porprismacoviruses are highlighted. d) Bayesian inference tree was constructed using MrBayes v3.2 based on amino acid sequences of Rep protein and visualized with iTOL. The viruses identified in this study are highlighted within the tree. Scale bar indicates the amino acid substitutions per site.

A total of 7 viruses belonging to the Smacoviridae were identified in this study, with genome lengths ranging from 1,421 to 2,427 bp and an average GC content of 50.85%. The complete genome sequence of gps222sma6 (accession number: MW183071) was obtained, which have an ambisense genome organization containing two major inversely arranged ORFs, encoding the rep and cap gene. A stem-loop structure with a conserved nonanucleotide sequence “TAGTGTTAC” motif in the loop, located between the 3′-ends of the two main ORFs (Fig. 4a). Phylogenetic analysis of the complete smacovirus Rep proteins showed them to be distinct from other CRESS-DNA viral families such as Circoviridae and Geminiviridae. As shown in Fig. 4d, all seven smacoviruses discovered in this study clustered together with the members of the genus *Porprismacovirus*. Members of the genus *Porprismacovirus* exhibited two major clusters. Cluster I was comprised of smacoviruses in cloacal samples from *Syrmaticus reevesii*, *Cygnus columbianus*, and *Chrysolophus pictus*. Cluster II included smacoviruses from *Pavo cristatus*, *Syrmaticus ellioti*, and *C. pictus*. Strains swn66sma1 and gps222sma6 were rather divergent from all other porprismacoviruses with <55% aa similarity with the closest porprismacoviruses mg8_345 (QIR82281), mg5_1444 (QIR82273), and Porcine-associated porprismacovirus 8 (YP_009054991). Identity analysis of the complete genome sequence of gps222sma6 showed a similarity of 49.56% to the closest virus (accession number: MH111079), which is below the species demarcation threshold of 77%, suggesting that gps222sma6 represents a novel species. Based on the identity matrix of the Rep protein shown in Fig. 4c, it can be observed that, except for the *Molossus molossus associated porprismacovirus 1* (accession number: UJO02102) discovered in bats in Argentina, which shares more than 90% identity with gps222sma2 (accession number: MW183070) from this study, the identities between the other six smacoviruses and other viruses are ranged from 30% to 70%. Pairwise comparison of all porprismacovirus Rep proteins showed sequence identities as low as 30%, reflecting a high level of genetic diversity. Conserved RCR and S3H motif analysis of the *Porprismacovirus* genus revealed that, except for blp211sma3 (accession number: MW183073) and gps215sma1 (accession number: MW183069), which lacked Motif I and Walker C due to incomplete Rep protein, the remaining five species exhibited intact conservation motifs (Fig. 4b).

### Identification of Novel Viruses of the Family Genomoviridae

The majority of Genomoviridae family members were discovered by metagenomic technique. At present, viruses in this family could be divided into ten genera (Varsani and Krupovic 2021), *Gemycircularvirus*, *Gemyduguivirus*, *Gemykroznavirus*, *Gemykibivirus*, *Gemygorvirus*, *Gemykolovirus*, *Gemytondvirus*, *Gemytripvirus*, and *Gemyvongvirus*, are nonenveloped, and a contain a ssDNA genome ranging from about 1.4 to 2.4 kb in size.

**Fig. 5.**
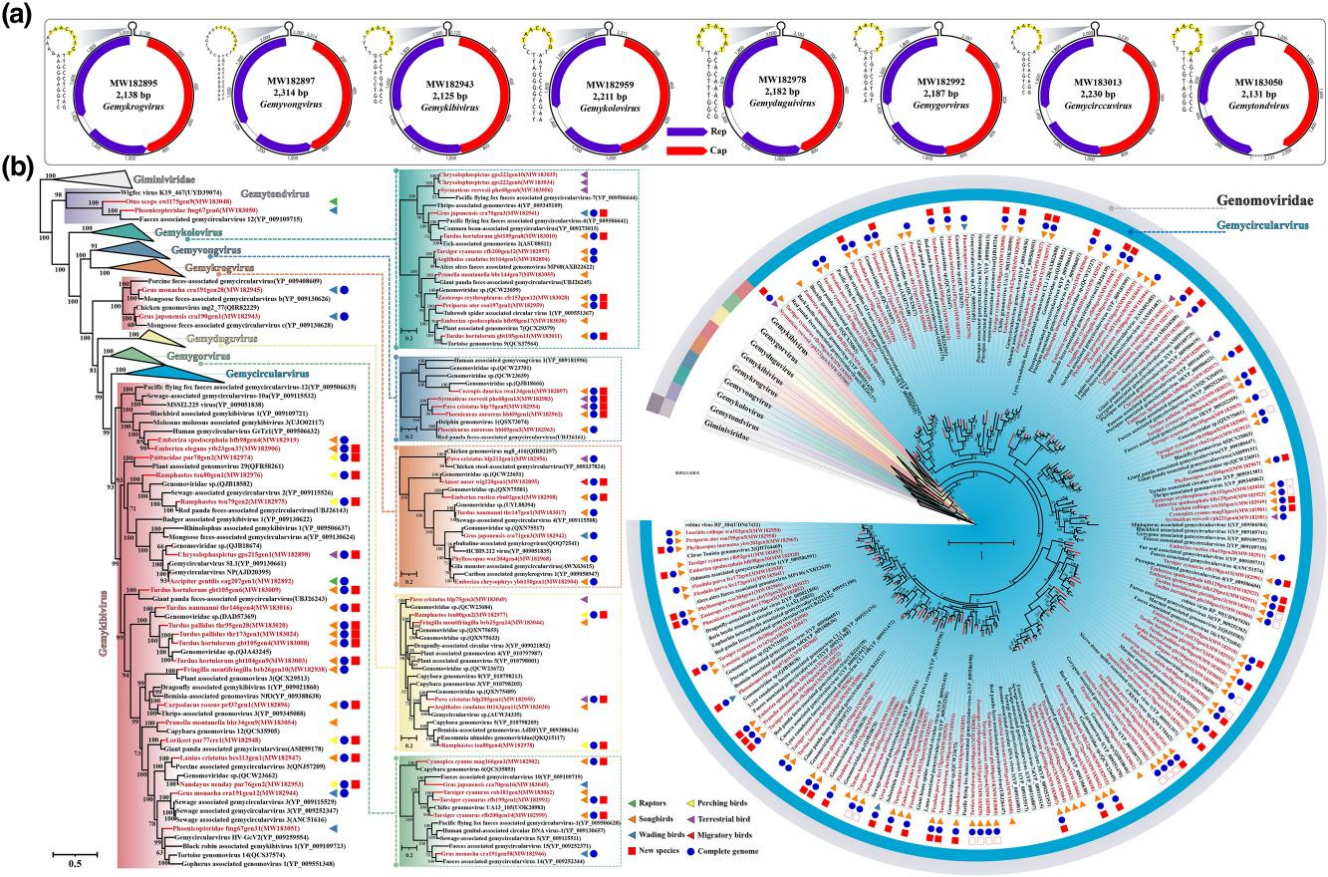
Identification of novel viruses of the family Genomoviridae. a) Predicted genome organization of MW182895 (*Gemykrogvirus*), MW182897 (*Gemyvongvirus*), MW182943 (*Gemykibivirus*), MW182959 (*Gemykolovirus*), MW182978 (*Gemyduguivirus*), MW182992 (*Gemygorvirus*), MW183013 (*Gemycircularvirus*), and MW183050 (*Gemytondvirus*). b) Bayesian inference tree was constructed using MrBayes v3.2 based on amino acid sequences of Rep protein and visualized with iTOL. The viruses identified in this study are highlighted within the tree. Scale bar indicates the amino acid substitutions per site.

In this study, 167 virus sequences belonging to 8 genera (*Gemycircularvirus*, *n* = 105; *Gemykibivirus*, *n* = 24; *Gemykolovirus*, *n* = 12; *Gemykrogvirus*, *n* = 11; *Gemyduguivirus*, *n* = 6; *Gemygorvirus*, *n* = 6; *Gemytondvirus*, *n* = 2; *Gemyvongvirus*, *n* = 1) of the family Genomoviridae had been identified. The novel members of the family Genomoviridae had an approximately 2.1 kb genome with a similar ambisense genomic organization. The genomovirus genomes were predicted to contain two ambisense ORFs encoding Rep protein and Cap protein. Furthermore, introns have been identified within the ORFs on several genomovirus genomes, whereas they have not been found in members of the family Smacoviridae and genus *Circovirus*. The intergenic region between 5′-ends of two major ORFs contained a stem-loop motif. On the apex of the loop, the conserved nonamer sequence 5′- TAWWDWRN -3′ is present (Fig. 5a). Phylogenetic analyses of 167 novel genomoviruses were conducted with 188 reference sequences of viruses in the family of Genomoviridae and 3 outgroup sequences in the family of Geminiviridae available in GenBank. As shown in Fig. 5b, the 168 viruses identified in this study clustered together with the 8 genera of Genomoviridae, and each branch had high posterior probabilities. Genomoviruses originating from songbirds are present in all seven genera. Interestingly, among the 105 newly identified viruses belonging to the genus *Gemycircularvirus*, 97 of them were derived from songbirds. According to the species demarcation criteria proposed by the ICTV, two genomoviruses must share >78% genome-wide nucleotide sequence identity to be classified as the same species (Krupovic et al. 2016). To further explore the presence of novel viral species within these groups, we conducted whole-genome sequence identity analysis on 132 viruses with complete circular genomes. The results (supplementary fig. S4, Supplementary Material online) revealed that among the 84 avian-related gemycircularviruses, 17 showed sequence identities above 78% with currently known viruses, while the remaining 67 viruses, with lower similarities to known viruses, potentially represent 58 new species. Among the 22 avian-related gemykibiviruses, there may be 16 new virus species. Additionally, the four avian-related gemygorviruses may represent three new virus species. The avian-related gemyduguiviruses (*n* = 3) and gemyvongvirus (*n* = 1) showed identities below 78% to known viruses, potentially indicating the presence of four distinct new virus species. Among the 11 avian-related gemykrogviruses, there may be 5 new virus species, and among the 7 avian-related gemykoloviruses, there may also be 5 new virus species.

### Identification of Novel Viruses of Unclassified CRESS-DNA Viruses

A total of 100 unclassified CRESS-DNA viruses were identified, among which 45 possessed complete circular genomes. These eukaryotic CRESS-DNA viruses are minimalists: their small circular genomes (<7.0 kb) encode fewer than 8 proteins, including the unique Rep and Cap proteins. Another significant feature of CRESS-DNA virus genomes is a conserved replication origin, marked by a predicted nine-nucleotide motif at the top of a stem-loop structure. The presence of the Cap protein distinguishes CRESS-DNA viruses from CRESS-DNA satellites or replicons, such as members of the Alphasatellitidae family.

**Fig. 6.**
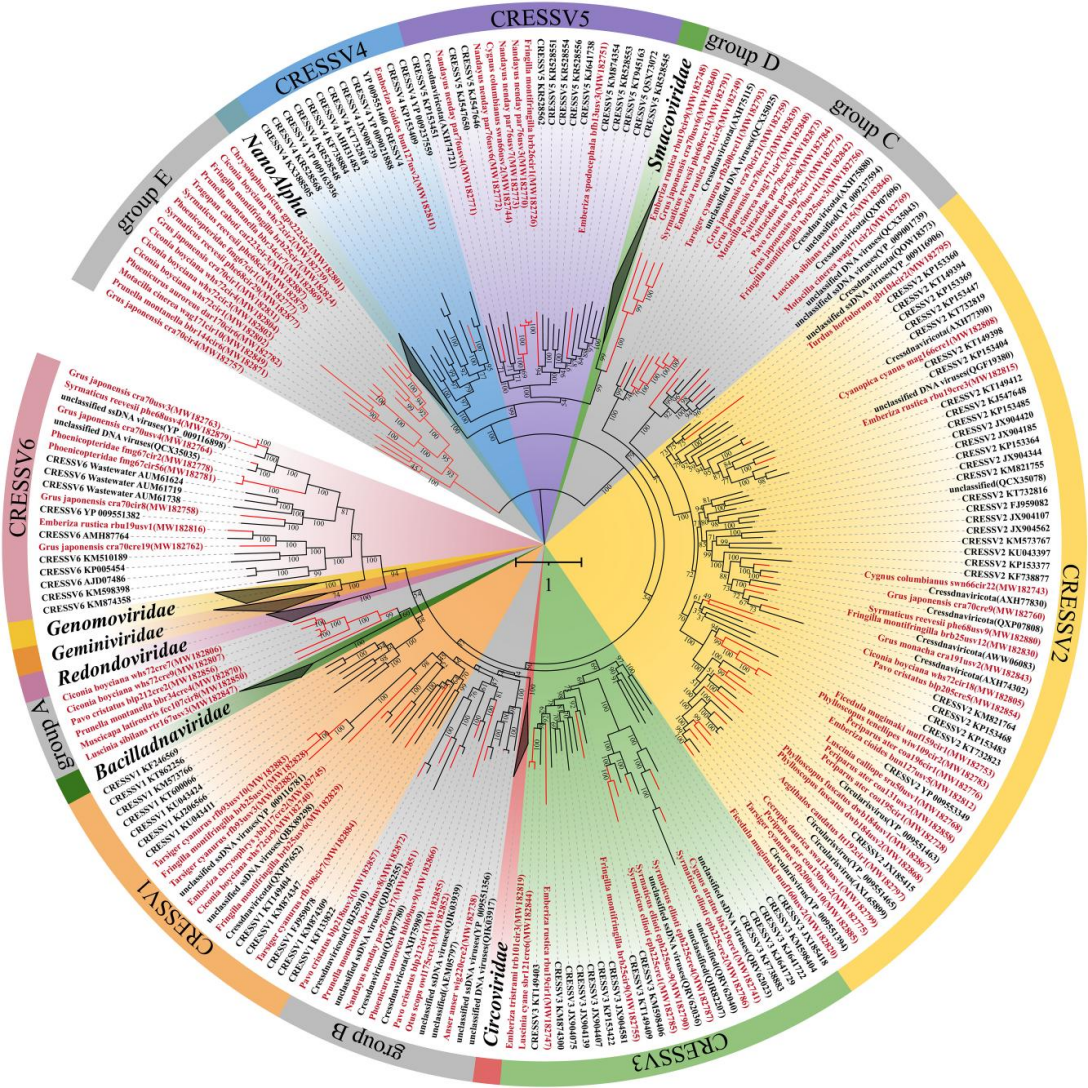
Identification of novel viruses of unclassified CRESS DNA viruses. Bayesian inference tree was constructed using MrBayes v3.2 based on amino acid sequences of Rep protein and visualized with iTOL. The viruses identified in this study are highlighted within the tree. Scale bar indicates the amino acid substitutions per site.

In this study, the Rep proteins of the 63 identified CRESS-DNA viruses share less than 70% amino acid similarity with sequences in NCBI (supplementary table S2, Supplementary Material online). Previous research has suggested that Rep proteins of CRESS-DNA viruses may have evolved from the HUH endonuclease gene of various bacterial and archaeal plasmids (Kazlauskas et al. 2019). Therefore, we conducted a phylogenetic analysis of retrieved Rep amino acid sequences from diverse organisms. In the resultant phylogenetic tree (Fig. 6), all previously established families of CRESS-DNA viruses and six groups of CRESSV1-6 are recovered as monophyletic with high statistical support. Fifty-six bird-associated unclassified CRESS-DNA viruses belong to CRESSV1-6. Our analysis also revealed five groups of unclassified CRESS-DNA virus groups. These groups were tentatively labeled groups A to E. In the phylogenetic tree, members of group A and group D branched with bacilladnaviruses and smacoviruses, respectively, whereas group B and group C showed stronger affinity to the corresponding domains of CRESSV1 and CRESSV1, respectively. In contrast, members of the group E were more divergent and generally branched separately from the other virus groups. Hence, groups A to E may represent new families of CRESS-DNA viruses. However, further investigation, such as an analysis of the corresponding capsid proteins, is required to validate this statement and will be described elsewhere.

**Fig. 7.**
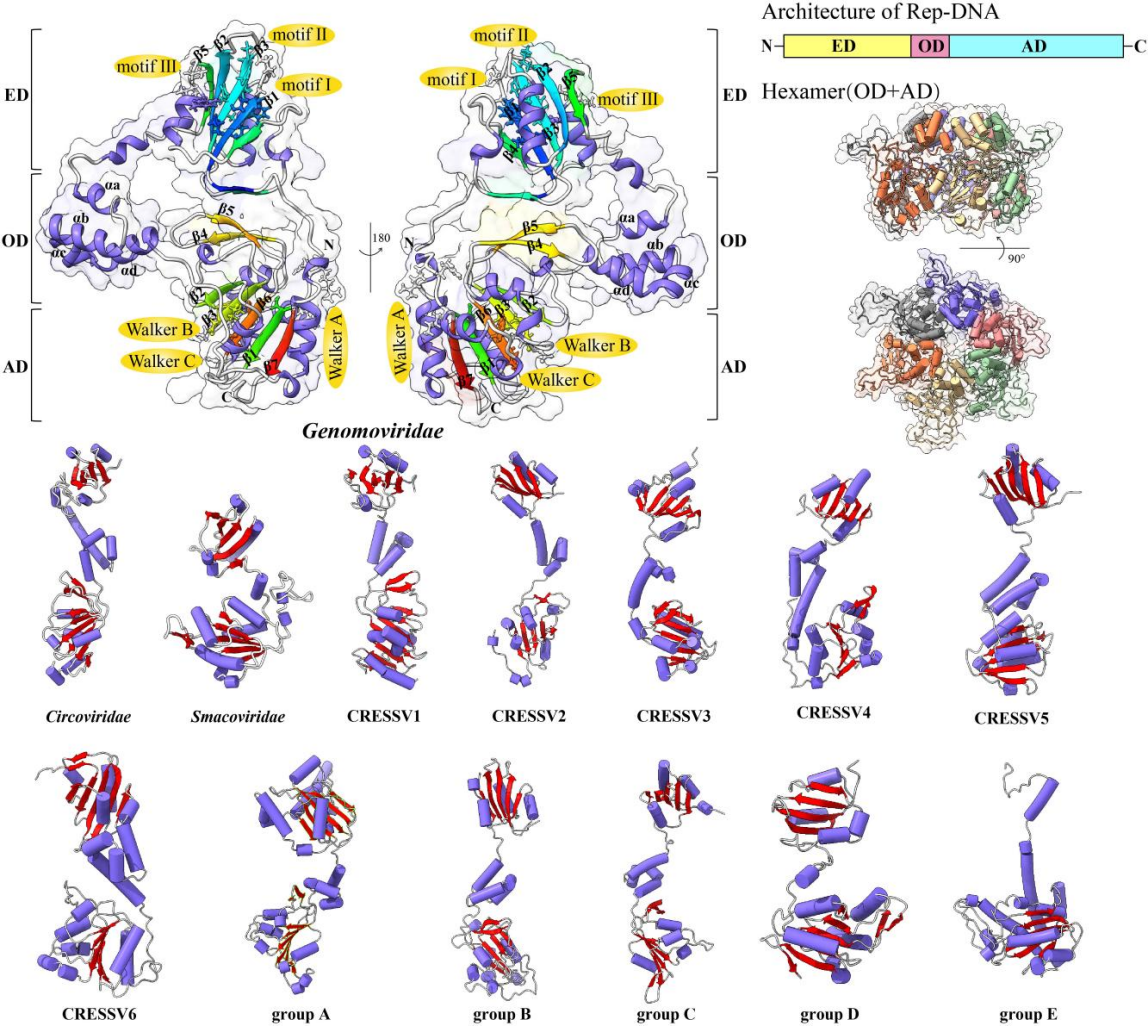
Tertiary structure comparison of the Rep protein in CRESS DNA viruses. Top left: ribbon cartoon representation of the genomovirus. Ribbon cartoons for each member of the 13 groups are distinguished based on their secondary structure. Top right: schematic of the Rep protein showing the ED, OD, and AD. Orthogonal views of the 3D map of the Rep protein are presented, showing both top and side perspectives. The six subunits are consistently distinguished. Figure generated using UCSF Chimera.

Using AlphaFold2, we predicted the protein structure to determine the 3D configuration of the Rep protein in CRESS-DNA viruses for this study (Fig. 7). The Rep protein is a hexameric molecule comprising three domains: the N-terminal HUH endonuclease domain (ED), the central oligomerization domain (OD), and the C-terminal ATPase domain (AD). The ED is responsible for the cleavage and ligation of ssDNA, the OD is pivotal for hexamer formation, and the AD interacts with both ssDNA and dsDNA, aiding in translocation along ssDNA while maintaining contact with the phosphate backbone (Tarasova et al. 2021). Upon observing the 3D models of the Rep protein, it became evident that structural differences exist among Rep proteins from various virus types. These variations include the number of β-sheets in both the ED and AD, the length of the OD, and the quantity of α-helices in the OD. Further investigation is needed to determine if these topological differences regulate the function of ED or influence hexamer formation. Predicting the Rep protein structure forms the basis for visualizing the initial stages of RCR.

## Discussion

We report 284 novel genomic sequences of CRESS-DNA viruses from cloacal swabs of wild and breeding bird specimens: 10 novel viruses belonging to the family Circoviridae, 7 novel representatives of the genus *Porprismacovirus* within the family Smacoviridae, 167 novel viruses belonging to the family Genomoviridae, and 100 unclassified CRESS-DNA viruses.

The family Circoviridae currently comprises 149 species, the majority of which have been identified in healthy hosts and are typically characterized by asymptomatic infection, without causing any tissue or organ damage or other diseases, such as PCV1 and most members of the genus *Cyclovirus* derived from parasitic arthropods. PCV1, the first member of the genus *Circovirus*, was discovered in 1974 as a contaminant of a porcine kidney cell line (Todd 2000). However, serological studies showed that the virus was widespread but nonpathogenic in pig populations in Canada and the United Kingdom, which led to limited attention. In contrast, two other members of the *Circovirus* genus, PCV2 and beak and feather disease virus (BFDV), are well known in the veterinary field. PCV2 is associated with clinical manifestations in pigs known as postweaning multisystemic wasting syndrome, while BFDV causes severe loss of feathers and deformities of the beak and claws in psittacine birds. While there is currently no definitive evidence linking cyclovirus infection to disease, certain species have been identified in humans presenting with paralysis (Smits et al. 2013), pneumonia (Prades et al. 2021), encephalitis, and diarrhea (Phan et al. 2015). Based on the widespread distribution of cycloviruses in invertebrates (arthropods) and the phylogenetic relationships between cyclovirus sequences from vertebrates and arthropods, we speculate that the two cycloviruses (cra70cir15 and rbf133cir1) identified in this study are primarily arthropod-infecting viruses. To date, smacoviruses have been identified by metagenomic analysis in a wide range of animal fecal samples, including insect samples and livestock serum (Cheung et al. 2013; Kazlauskas et al. 2017; Altan et al. 2018; Díez-Villaseñor and Rodriguez-Valera 2019). Smacoviruses have not yet been successfully cultured in laboratory cell lines, and no definite host has been identified; however, a research (Díez-Villaseñor and Rodriguez-Valera 2019) has suggested that methanogenic archaea associated with the intestinal tract could be the natural hosts based on identification of CRISPR spacers matching regions of smacovirus genomes. The term “*Porprismacovirus*” is derived from “Porcine and primate smacovirus,” indicating that viruses in this genus infect both pigs and primates. With the increasing discovery and identification of viruses in recent years, it has become evident that the family Smacoviridae is much larger than previously anticipated. From only 6 genera in 2018, it has now expanded to 12 genera in 2023. Furthermore, *Porprismacovirus* is not limited to pigs and primates but has also been found in other domestic animals, poultry, and even wild birds in this study. This highlights the remarkable diversity of smacoviruses and provides a foundation for investigating their potential public health significance through preliminary analysis of viral sequences. Genomoviruses have been identified from a variety of eukaryotic samples, including fungi, plants, and insects. They have also been amplified or detected from environmental samples such as sewage, wastewater, and sediments (Varsani and Krupovic 2017). These viruses have also been reported to be associated with human infections, although their pathogenic role has not been verified to date (Bezerra et al. 2020). In our study, a total of 167 genomoviruses were identified, among which 132 possessed a complete genomic structure. Based on phylogenetic analysis and sequence identity assessments, it was revealed that these may potentially represent 91 new species. This research significantly enriches our comprehension of genomovirus diversity and lays a solid groundwork for further investigations into their biology and implications. Members of the phylum Cressdnaviricota represent a highly diverse group of circular DNA viruses characterized by elevated nucleotide substitution rates. It is plausible that these viruses have evolved from the interaction between RNA viral capsid protein genes and bacterial plasmids on multiple independent occasions. The majority of these viruses were discovered through metagenomic approaches and remain uncultured, leading to limited understanding of their hosts and their impact on environmental diversity (Rosario, Dayaram, et al. 2012).

Here, we conducted an analysis of CRESS-DNA virus genomic structures, whole-genome sequence similarities, and evolutionary relationships among Rep proteins, culminating in the identification of 284 new viruses belonging to the phylum Cressdnaviricota. Among these identified viruses, geminiviruses were the most prevalent, followed by unclassified CRESS-DNA viruses, circoviruses, and smacoviruses. In the case of geminiviruses from the family Genomoviridae, the genera *Gemycircularvirus* and *Gemykibivirus* dominated, with reports suggesting their presence in sewage, thrips, chickens, bats, plasma of HIV-positive patients, and pericardial fluids of patients with pericarditis. These studies underscore the wide distribution and potential pathogenicity of geminiviruses. However, it is worth noting that although geminiviruses have been found in various organisms, the definitive host species for members of this family remain unverified. Previous research has indicated a correlation between the evolutionary relationships of members of the *Circovirus* genus and their hosts. Nevertheless, the newly identified circular viruses from wild bird intestines did not cluster with known avian circoviruses but instead formed distant branches with fish and mammalian counterparts. Additionally, we identified eight groups of previously unreported unclassified CRESS-DNA viruses. A thorough analysis of these viral clusters could offer valuable insights into the origin and evolution of these “classic” CRESS-DNA viral groups.

The most astonishing finding of this study was the identification of a diverse array of eukaryotic viruses in bird feces, potentially reflecting the diversity of animals and plants consumed by birds. Likewise, representatives of invertebrate and even plant-infecting viruses that are locally transmitted in the ecosystem were found in bird fecal samples (Shan et al. 2022). It remains unclear whether the viral particles present in bird feces were infectious, as described previously in the case of pepper mild mottle virus, which passed through the human gastrointestinal tract and retained its infectivity (Zhang et al. 2006).

In summary, in this report, we describe several novel members of the phylum Cressdnaviricota. This work provides the first data set of CRESS-DNA viruses from wild and breeding bird in mainland China, and it contributes to a better understanding of the evolution and genetic diversity of these viruses. The identification of CRESS-DNA viruses in avian species poses several inquiries that warrant exploration in future studies. Addressing these questions is essential to unravel the factors influencing the spread of this virus. Moreover, additional investigations are warranted to assess the potential impact of these recently characterized viruses on bat colonies. The data presented in this study will facilitate such inquiries by providing sequence information for in silico analysis and guiding the design of consensus primers for future research.

## Materials and Methods

### Sample Collection and Preparation

A total of 3,404 cloacal swabs of wild and breeding bird specimens were collected for a previously published virome study from 5 different provinces in China (Fig. 1a) from 2018 to 2019 (Shan et al. 2022). Ethical approvals were given by the Ethics Committee of Chinese Academy of Agricultural Sciences with the reference number SVRI2017091, the Ethics Committee of Jiangsu University with the reference number 2018ujs18023, and the Ethics Committee of Key Laboratory of Wildlife Diseases and Biosecurity Management of Heilongjiang Province with the reference number WDBM2018-023. All specimens were shipped on dry ice. Cloacal swabs specimens were resuspended individually in 0.5 mL phosphate-buffered saline and vortexed at 1,800 rpm for 5 min and centrifuged for 10 min at 15,000 × *g*; the supernatant was then collected in a microcentrifuge tube and stored at −80 °C. About 0.1 mL supernatant of each cloacal swab specimen from the same bird species was added to sample pools (supplementary table S1, Supplementary Material online). Subsequently, the supernatant was filtered through a 0.45-μm filter (Millipore, Darmstadt, Germany) to remove eukaryotic, giant viruses and bacterial cell-sized particles (Lu et al. 2024).

### Viral Metagenomic Analysis

The filtrates enriched in viral particles were then treated with a cocktail of DNase, RNase, benzonase, and Baseline-ZERO to digest unprotected nucleic acid at 37 °C for 90 min (Zhang et al. 2014). Total nucleic acids were then extracted using QIAamp MinElute Virus Spin Kit (Qiagen) according to the manufacturer's protocol. Two hundred and thirty-eight libraries were then constructed using a Nextera XT DNA Sample Preparation Kit (Illumina) and sequenced using the Illumina MiSeq platform with 250 base paired-ends with dual barcoding for each individual sample or sample pool. The information about each library is shown in supplementary table S1, Supplementary Material online. For bioinformatics analysis, paired-end reads of 250 bp generated by MiSeq were debarcoded using vendor software from Illumina. An in-house analysis pipeline running on a 32-node Linux cluster was used to process the data. Clonal reads were removed, and low-sequencing-quality tails were trimmed using a Phred quality score of 10 as the threshold. Adaptors were trimmed using the default parameters of VecScreen, which is NCBI BLASTn with specialized parameters designed for adapter removal. The cleaned reads were de novo assembled within each barcode using the ENSEMBLE assembler (Deng et al. 2015). Contigs and unassembled reads are then matched against a customized viral proteome database using BLASTx with an E-value cutoff of <10^−5^, where the virus BLASTx database was compiled using NCBI virus reference proteome (https://ftp.ncbi.nlm.nih.gov/refseq/release/viral/) to which was added viral protein sequences from NCBI nr fasta file (based on annotation taxonomy in Virus Kingdom). Candidate viral hits are then compared to an in-house nonvirus nonredundant (NVNR) protein database to remove false positive viral hits, where the NVNR database was compiled using nonviral protein sequences extracted from NCBI nr fasta file (based on annotation taxonomy excluding Virus Kingdom). Contigs without significant BLASTx similarity to viral proteome database are searched against viral protein families in vFam database (Skewes-Cox et al. 2014) using HMMER3 (Eddy 2009; Johnson et al. 2010; Finn et al. 2011) to detect remote viral protein similarities.

### Analysis of the Sequence

For assembly of the virus genomes, the contigs showing significant BLASTx similarity to parvoviruses were selected (Kearse et al. 2012). The contigs with consensus sequence length >500 bp were subjected to further analysis where the individual contig was used as reference for mapping to the raw data of its original barcode using the Low Sensitivity/Fastest parameter in Geneious. Those prolonged contigs that had the major Rep protein and capsid (Cap) protein, as well as some contigs that only had a nonstructural protein, were included in this study. The contig only had a putative Cap protein that was not showed in this study. Splice sites were identified through the application of the Neural Network from the Berkeley Drosophila Genome Project (Reese et al. 1997; Dogan et al. 2007). Introns within the Rep-encoding region were manually recognized in instances where multiple ORFs exhibited alignment with Reps. Predicted spliced Reps were included in the analysis only if they encompassed all conserved motifs associated with known eukaryotic CRESS-DNA viral Reps, specifically the RCR and helicase motifs. These spliced Reps were subsequently employed in phylogenetic comparisons.

The search for protein homologies was made by BLAST programs at the NCBI website (http://www.ncbi.nlm.nih.gov/Blast.cgi) against the nonredundant protein database. The potential origin of replication (ori) for each genome was determined by identifying a canonical nonanucleotide motif (NANTATTAC) (Rosario, Duffy, et al. 2012) and confirming the predicted stem-loop structures using Mfold (http://www.unafold.org/mfold/applications/dna-folding-form.php). Constraints were applied to prevent hairpin formation within the nonanucleotide motif, and the folding temperature was set at 17 °C (Zuker 2003).

### Phylogenetic Analysis

Full CRESS-DNA viral genomes were downloaded from GenBank. For eukaryotic CRESS-DNA viral genomes, the Rep and Cap protein coding regions were extracted from each genome. Introns within the Rep coding region were identified manually when there were multiple ORFs with matches to Reps. Outgroups were used, when possible; otherwise, the trees were midpoint rooted. Then, Bayesian inference trees were constructed with MrBayes v3.2 (Ronquist et al. 2012). During MrBayes analysis, we set “prset aamodelpr=mixed” for the phylogenetic analysis using amino acid sequences, which allows the program to utilize the ten built-in amino acid models. The Markov chain was run for a maximum of 1 million generations, in which every 50 generations were sampled and the first 25% of Markov chain Monte Carlo samples were discarded as burn-in, and the tree was visualized with iTOL (https://itol.embl.de/) (Letunic and Bork 2021). Color-coded distance matrix analysis was performed with Sequence Demarcation Tool v1.2 (Muhire et al. 2014).

## Supplementary Material

evae206_Supplementary_Data

## Data Availability

The nucleotide sequences were deposited in the GenBank database, and the accession numbers are shown in supplementary table S2, Supplementary Material online. The sequence raw data of bird cloaca samples were deposited into the NCBI Sequence Read Archive under accession number PRJNA600556.
