# Supplementary material for: Unveiling the Virome of Wild Birds: Exploring CRESS-DNA Viral Dark Matter
Source: Genome Biol Evol. 2024 Sep 27;16(10):evae206. doi: 10.1093/gbe/evae206 (PMC11463337; doi:10.1093/gbe/evae206)

Supplementary Fig. 1

Species accumulation curves of 228 pools. The light blue shaded area represented the confidence interval of the curve.

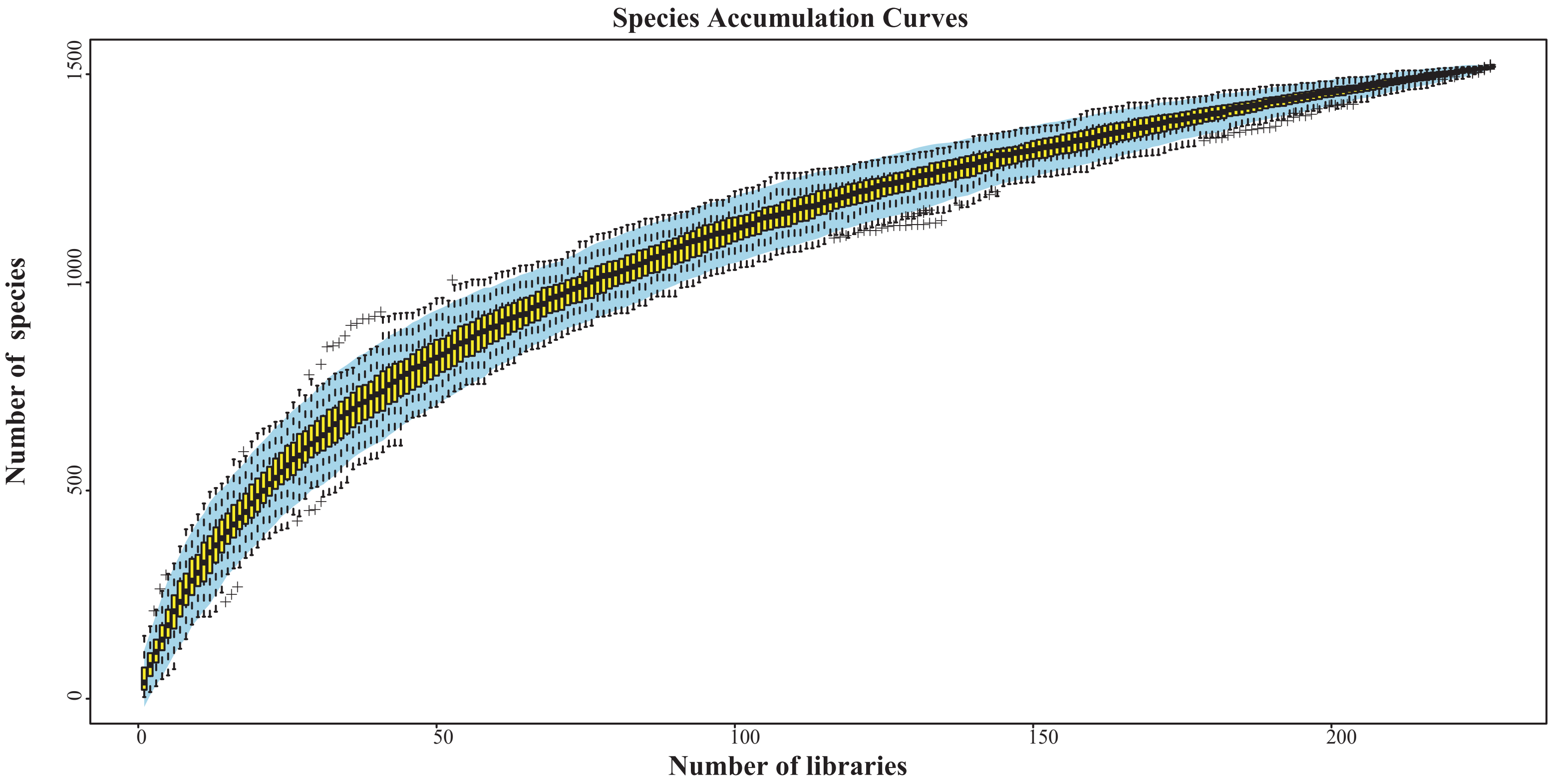

Supplementary Fig. 3

The distribution of dominant viruses at different taxonomic levels (viruses with relative abundance > 1% of the total sequences are shown in the larger left pie chart, with high abundance viruses indicated in red font and low abundance viruses in black font).

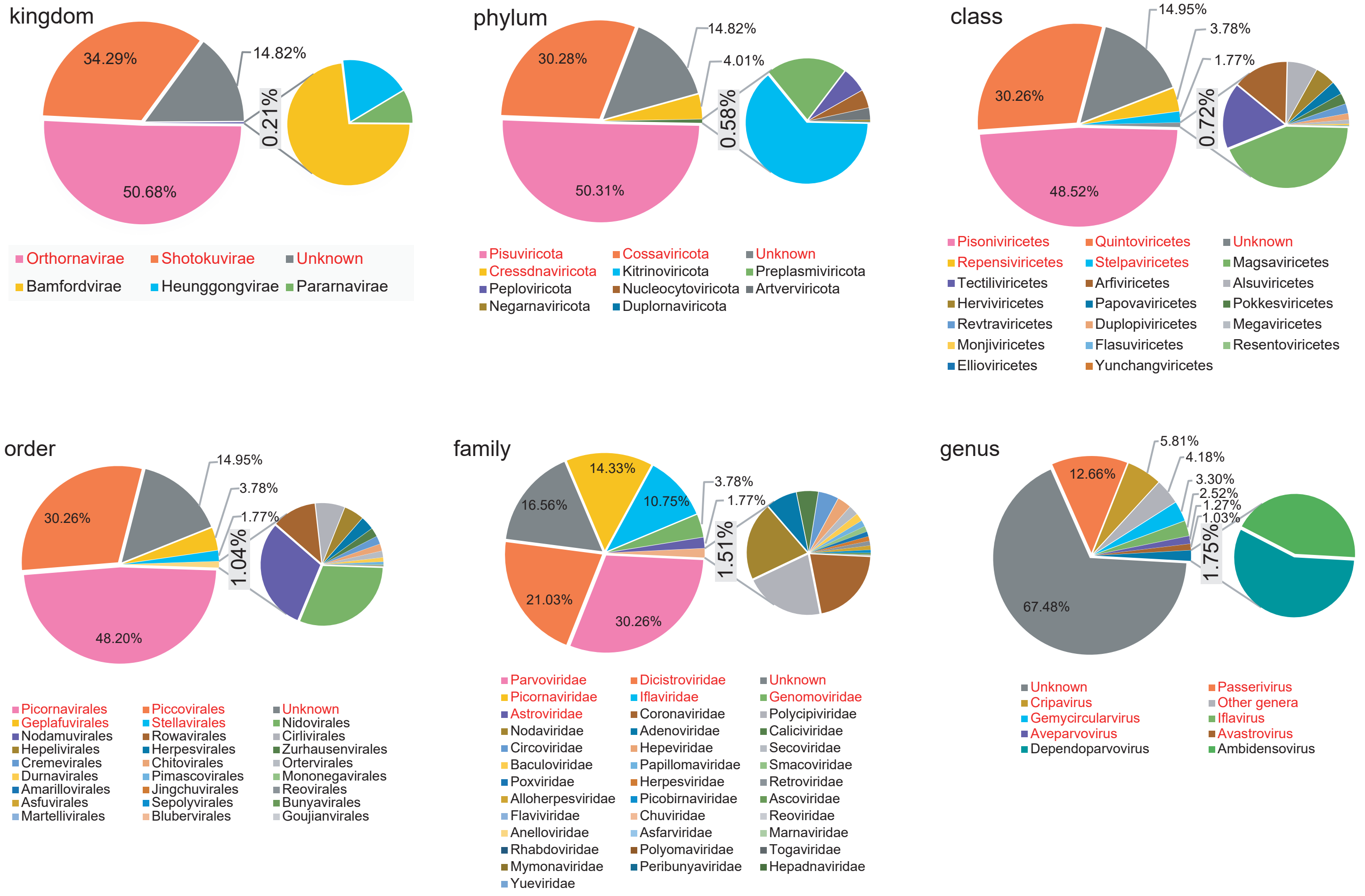

Supplementary Fig. 2

Taxonomy visualization. A taxonomy tree visualized by GraPhlAn. The inner ring with blue squares represents the genus detected in the cloacal swabs of birds with a relative abundance greater than 0.1%, while the outer ring with yellow triangles represents the genus detected in the cloacal swabs of birds with a relative abundance lower than 0.1%.

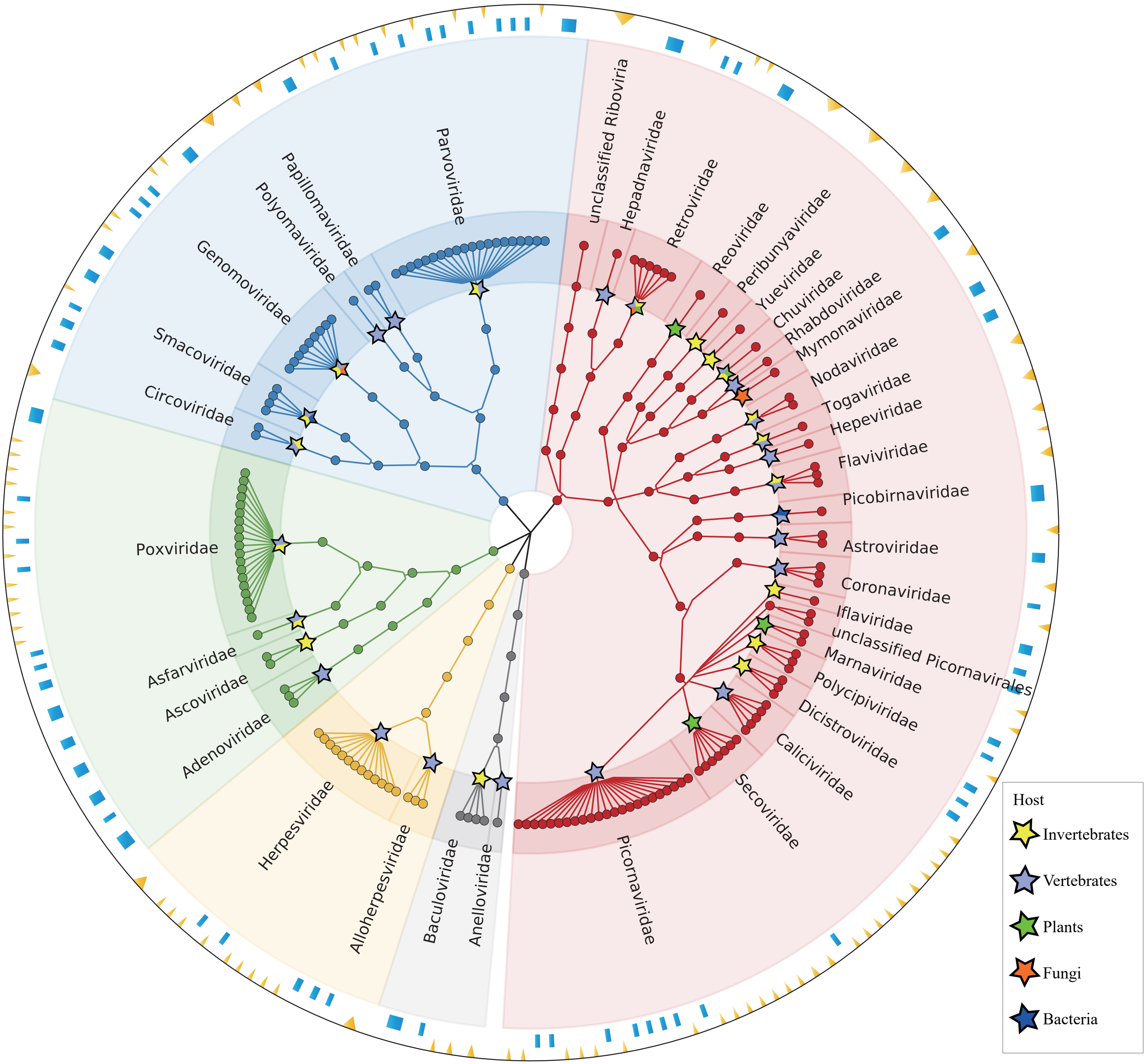

Supplementary Fig. 4

Pairwise sequence comparison produced with full-genome sequences of members of *Genomoviridae* family.

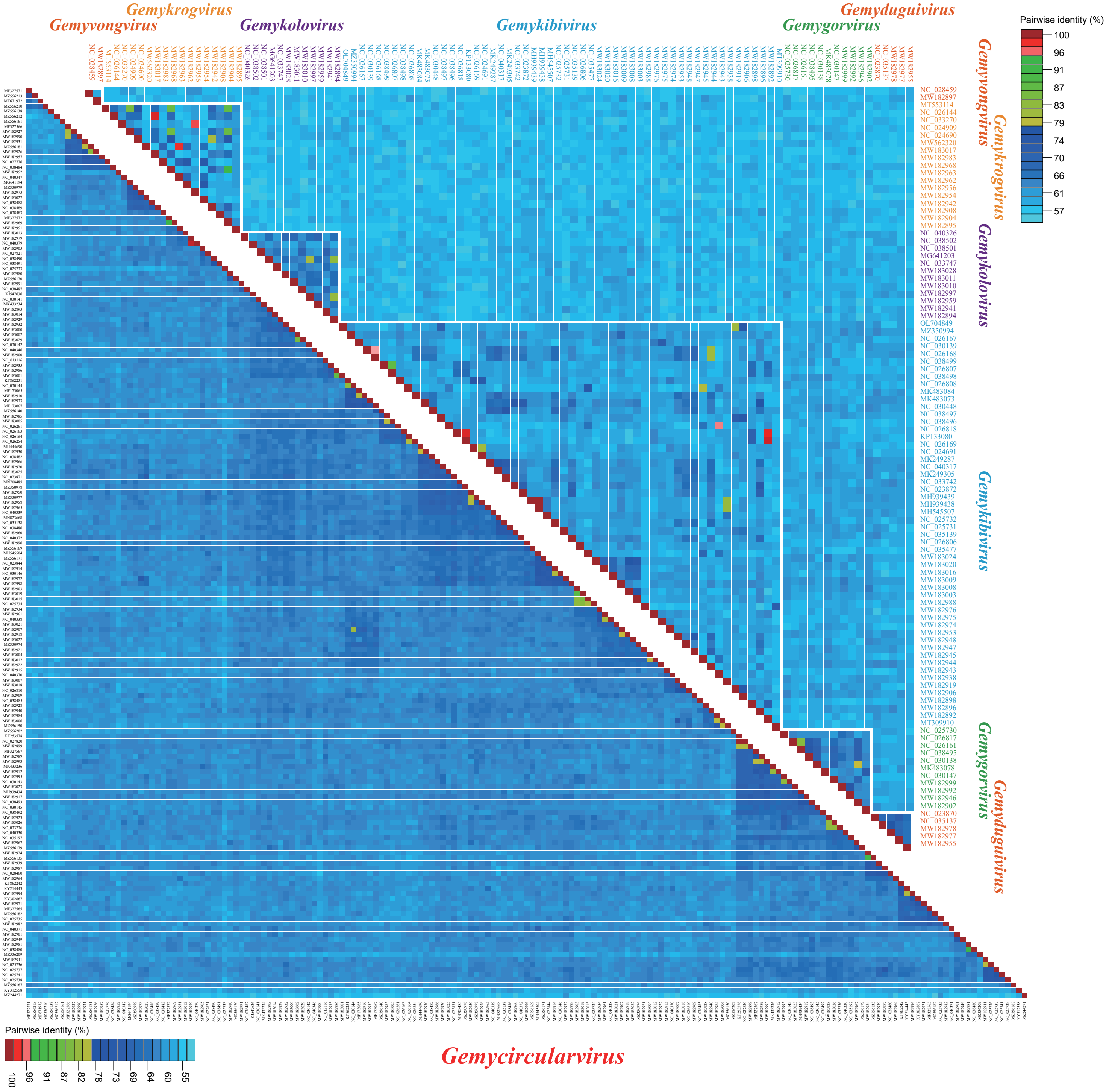

Supplement: evae206_Supplementary_Data [file evae206_supplementary_data.zip › Supplementary Fig.pdf]
